# Zooplankton From a Reef System Under the Influence of the Amazon River Plume

**DOI:** 10.3389/fmicb.2018.00355

**Published:** 2018-03-01

**Authors:** Sigrid Neumann-Leitão, Pedro A. M. C. Melo, Ralf Schwamborn, Xiomara F. G. Diaz, Lucas G. P. Figueiredo, Andrea P. Silva, Renata P. S. Campelo, Mauro de Melo Júnior, Nuno F. A. C. Melo, Alejandro E. S. F. Costa, Moacyr Araújo, Dóris R. A. Veleda, Rodrigo L. Moura, Fabiano Thompson

**Affiliations:** ^1^Departamento de Oceanografia, Universidade Federal de Pernambuco, Recife, Brazil; ^2^Departamento de Biologia, Universidade Federal Rural de Pernambuco, Recife, Brazil; ^3^Instituto Sócioambiental e Recursos Hídricos, Universidade Federal Rural da Amazônia, Belem, Brazil; ^4^Universidade Federal do Rio de Janeiro – Instituto de Biologia e SAGE/COPPE, Rio de Janeiro, Brazil; ^5^Rede Brasileira de Pesquisas sobre Mudanças Climáticas Globais – Rede CLIMA, São José dos Campos, Brazil

**Keywords:** zooplankton, Amazon plume, reef system, copepod functional trait, diversity

## Abstract

At the mouth of the Amazon River, a widespread carbonate ecosystem exists below the river plume, generating a hard-bottom reef (∼9500 km^2^) that includes mainly large sponges but also rhodolith beds. The mesozooplankton associated with the pelagic realm over the reef formation was characterized, considering the estuarine plume and oceanic influence. Vertical hauls were carried out using a standard plankton net with 200 μm mesh size during September 2014. An indicator index was applied to express species importance as ecological indicators in community. Information on functional traits was gathered for the most abundant copepod species. Overall, 179 zooplankton taxa were recorded. Copepods were the richest (92 species), most diverse and most abundant group, whereas meroplankton were rare and less abundant. Species diversity (>3.0 bits.ind^-1^) and evenness (>0.6) were high, indicating a complex community. Small holoplanktonic species dominated the zooplankton, and the total density varied from 107.98 ind. m^-3^ over the reef area to 2,609.24 ind. m^-3^ in the estuarine plume, with a significant difference between coastal and oceanic areas. The most abundant copepods were the coastal species *ithona plumifera* and *Clausocalanus furcatus* and early stages copepodites of Paracalanidae. The holoplanktonic *Oikopleura*, an important producer of mucous houses, was very abundant on the reefs. The indicator species index revealed three groups: (1) indicative of coastal waters under the influence of the estuarine plume [*Euterpina acutifrons, Parvocalanus crassirostris, Oikopleura (Vexillaria) dioica* and Hydromedusae]; (2) characterized coastal and oceanic conditions (*Clausocalanus*); (3) characterized the reef system (*O. plumifera*). Two major copepods functional groups were identified and sorted according to their trophic strategy and coastal-oceanic distribution. The species that dominated the coastal area and the area over the rhodolith beds are indicators of the estuarine plume and are mixed with species of the North Brazil Current. These species practically disappear offshore, where occur oceanic species commonly found in other oligotrophic tropical areas. This ecosystem shows a mixture of estuarine, coastal and oceanic communities coexisting in the waters over the Amazon reefs, with no significant differences among these areas. However, the MDS clearly separated the communities along the salinity gradient in the plume.

## Introduction

Coastal waters with a riverine influence are extremely productive, ecologically and societally valued ecosystems (Biber et al., 2005). These waters are subject to the increasing impacts of sedimentation and nutrient enrichment caused by human disturbances across many scales, imposing serious threats to ecosystems and human health (Paerl, 1997). There is already global concern about the consequences of anthropogenic activities in coastal and oceanic regions leading to innumerable impacts. These impacts are expected to be exacerbated by climate change (Paerl et al., 2005). Among the coastal environments that are subject to threats from anthropogenic changes, the Amazon is one of the least surveyed, although the freshwater outflow accounts for ∼ 18% of the global riverine discharge to the ocean (Milliman and Meade, 1983) and the massive sediment supply (10% of the world) (Martinez et al., 2009).

Given the overall importance of the coastal area of Amazonia and to understand the various complex interactions that link estuarine-costal-oceanic systems, two complementary multidisciplinary projects were proposed. NOVOBIOMA aimed to characterize the distribution, structure and biodiversity associated with the reef formations of the Brazilian North Coast (Amapá, Pará and Maranhão) and the major physical, chemical and biological properties of the water column overlying this Brazilian biome, which has been little studied to date (Moura et al., 2016). The Camadas Finas V project sought to characterize the dominant oceanographic mechanisms along the Amazon plume, from the estuary to coastal transport through the North Brazil Current. As an integral component of both projects, zooplankton represent a formidable challenge in terms of determining the ecological status and trends.

The role of zooplankton in the food web as energy transfer, linking bottom–up (e.g., phytoplankton) to top–down elements (e.g., fish, mammals) make it an important indicator of ecological conditions (Kiørboe, 2011), thus providing information for fisheries production and for local, regional and global biogeochemical cycles (Miyashita et al., 2009). In addition to this significant role in nutrient cycling and energy transport, zooplankton include the larval stages of some non-planktonic organisms, influencing the success of the future nektonic and benthic communities (Lenz, 2000), including those associated with the extensive carbonate system near the mouth of the Amazon beneath the river plume. They also play essential roles in biological pump, feeding in epipelagic waters and carrying carbon to meso-bathypelagic and bottom areas through fecal pellet and dynamic transfer via their diel vertical migrations (Steinberg et al., 2002, Turner, 2015, Conroy et al., 2016).

Zooplanktonic communities are strongly affected by the dynamics of abiotic processes (e.g., physical, chemical, and climate processes) that influence water masses in the marine milieu (Haury et al., 1990; Boltovskoy, 1999). The higher density and biomass of plankton found in physical structures such as estuarine plumes, biogenic reefs, eddies and frontal zones (Roman et al., 2001; North and Houde, 2003; Goldthwait and Steinberg, 2008; Flint et al., 2010; Hernández-León et al., 2013; Melo et al., 2016), have major effects on biogeochemical cycling and sustain higher-order consumers in trophic webs (Pauly and Christensen, 1995; Benoit-Bird and McManus, 2012). The Amazon River generates an offshore plume of up to 1.5 × 10^6^ km^2^ that extends to the western tropical North Atlantic (Coles et al., 2013; Goes et al., 2014), generating a physical and chemical gradient supporting diverse plankton communities (Carpenter et al., 1999; Foster et al., 2007; Goes et al., 2014).

In most tropical and subtropical marine waters, the zooplankton community is dominated by copepods, which occupy key trophic positions (Neumann-Leitão et al., 2008; Melo P.A.M.C. et al., 2014; Costalago et al., 2015). The functional diversity of copepods may link the system’s biological structure to ecosystem processes (Pomerleau et al., 2015). Considering that understanding how functional traits vary across space and time is important to elucidate fundamental ecological processes determining species diversity, community structure and ecosystem functioning (Bertelsmeier, 2017), identifying and describing the functional traits of copepods should increase our comprehension of their ecological role in the Amazon system.

The available zooplankton data for the Amazon coast are very limited because this is one of the areas in the Atlantic equatorial region with little available information (Boltovskoy, 1999; Conroy et al., 2016). The relevant reported zooplankton studies include those of Dahl (1894) and Björnberg (1963) on copepods; Vannucci and Queiroz (1963) and Jacob et al. (1966) on zooplankton biovolume; Paranaguá (1966) on general zooplankton taxa; Calef and Grice (1967), related to the total zooplankton volume and copepod abundance, in addition to cladoceran and decapod abundances associated with the low-salinity plume; Alvariño (1969) on chaetognaths; Montú (1994) on amphipods; Melo N.F.A.C. et al. (2014) on the distribution of Luciferidae; and Conroy et al. (2016) on meso- and microzooplankton grazing in the Amazon River plume. Most of the available information on zooplankton abundance and the community composition in this region can be found in the gray literature, mainly consisting of abstracts from scientific congresses, post-graduation monographs and dissertations/theses.

Within this context, our hypothesis is that the reef and oceanic zooplankton is modified by the Amazon estuarine plume. Thus, the present study aims to investigate the structure and function of the mesozooplankton community over the recently rediscovered reef under the influence of the Amazon estuarine plume and the role of the plume on the zooplankton from coastal to oceanic waters, including retroflection effects. We highlight the composition and abundance of mesozooplankton, ecological descriptors (bioindicators and copepod species diversity, evenness and functional traits) in this unique carbonate reef system.

## Materials and Methods

### Study Area

The Amazonia Coast of Brazil extends from the mouth of the Parnaíba River to the mouth of the Oiapoque River. This coast has been subdivided into three sectors: the Guyanese Coast, Golfão Marajoára and Amazonian Eastern Coast (Coutinho, 1995). The width of the Amazon continental shelf (ACS) ranges from 100 km, at the mouth of the Parnaíba River, to 330 km, opposite the island of Marajó, and its slope tends to decrease with an increasing width and depth. The continental shelf break occurs in the range of 80 to 120 m (Coutinho, 1995).

Coastal currents represent one of the most important mechanisms that play a role in the forcing of the ACS (Molinari, 1982; Stramma, 1991). The Northern Brazil Current (NBC) forms from the bifurcation of the southern branch of the South Equatorial Current (sSEC) that moves northward as the North Brazilian Undercurent (NBUC). The NBC is also fed by the central branch of the South Equatorial Current (cSEC), carrying approximately 21 Sv at 1°S, and from that point, the North Brazil Current continues northwestward, carrying approximately 37 Sv just north of 5°S (Silveira et al., 1994).

The Amazon River discharges an average of 1.8 × 10^5^ m^3^ s^-1^ of fresh water onto the ACS (Forbes et al., 1991; Forbes and Liverman, 1996), representing 18% of the total fresh water discharged from all rivers into the ocean (Milliman and Meade, 1983). The freshwater discharge varies seasonally, with the maximum discharge of 2.5 × 10^5^ m^3^ s^-1^ occurring in May and the minimum of 1.2 × 10^5^ m^3^ s^-1^ occurring in November (Castro and Miranda, 1998). The plume of the Amazon River also extends northwestward, penetrating more than 1000 km in the North Atlantic (Gibbs, 1970; Müller-Karger et al., 1988). Lentz (1995) noted that from January to June, the plume of the Amazon River north of 5°N is located primarily west of 52°W, extending toward the Caribbean; and, that from August to October, approximately 70% of the water in the Amazon River plume is carried eastward via retroflection of the North Brazil Current, and the remaining 30% is diverted toward northwest to the Caribbean.

The Amazon reef area presents three main zones. (1) The northern zone, composed by shelf-edge reefs with abundant crustose calcareous algae (including rhodoliths), and sponges (*Clathria* sp., *Monanchora arbuscula, Oceanapia bartschi*). This zone contains a clear oxycline between the plume and sub-plume waters, with anaerobic metabolism in the plume and sulfate reduction/S oxidation in the sub-plume layers. (2) The central zone containing sand waves, where the same crustose calcareous algae and sponges occur. 3) The southern zone, characterized by low-relief patchy reefs with the dominance of scleractinian corals (Moura et al., 2016). The reef system is extensive, impoverished in terms of biodiversity, and presents unique functional attributes due to the plume influence.

### Sampling Strategy and Laboratory Procedures

**FIGURE 1:**
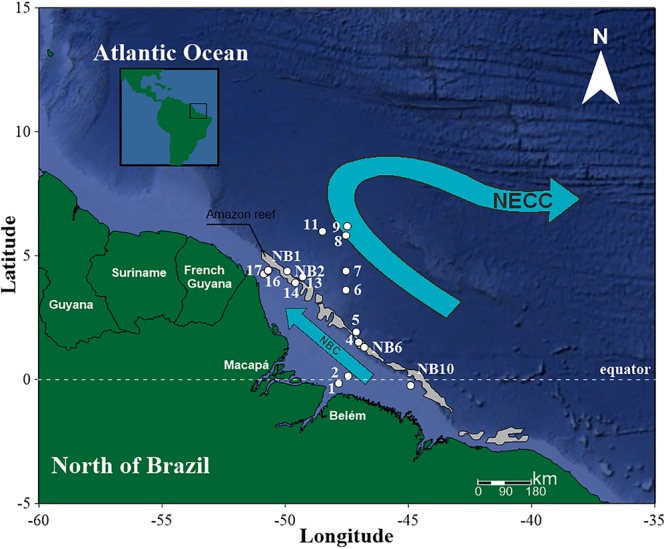
Studied area and stations in the Amazon mouth reef system and adjacent areas (estuarine plume and oceanic system) in September/2014. The arrows represent the main surface current systems flowing in the area. NBC, North Brazil Current; NECC, North Equatorial Counter-Current. The reef system is represented in gray, the continental-shelf in light blue, and the continental-slope and ocean basin in dark blue.

The survey was performed during a single campaign carried out from September 24 to 29 of 2014, along the Brazilian North Coast (Amapá, Pará and Maranhão) (**Figure 1**). In this campaign, two field projects were developed: NOVOBIOMA and Camadas Finas V, on board the research vessel HOc. Cruzeiro do Sul – H38 (DHN/Brazilian Navy).

The stations visited as part of NOVOBIOMA (NB1, NB2, NB6, and NB10, the reef area) and Camadas Finas V (St. 1 to St. 17) were sampled using a ring plankton net (200 μm mesh size), which was hauled vertically to the surface from a depth of 5–7 m from the bottom in the nearshore area and 150 m offshore of the shelf break. A flowmeter (Hydrobios, Kiel) was fitted onto the opening of the net. Samples were collected and preserved in a 4% buffered formalin-seawater solution. Sestonic biomass was estimated via the wet-weight method (Omori and Ikeda, 1984). No significant amounts of sediment were observed in the samples.

Zooplankton species were identified using the manuals produced by Trégouboff and Rose (1957) and Boltovskoy (1981, 1999), among others, and taxon counting considered the lowest taxonomic unit that it was possible to identify for each phylum. The scientific names followed the World Register of Marine Species (WORMS) database. A Motoda splitter was used to fractionate the samples into fractions of up to 1/64, provided that a minimum number of 300 individuals were analyzed. The samples that did not reach this minimum number of individuals were analyzed in their entirety. To analyze rare species, fractions that were larger than the initially analyzed fraction were examined.

Abiotic data (temperature and salinity) and chlorophyll-a were determined using a CTD. The thermohaline water mass were differentiated as follows: South Atlantic Central Water (SACW, temperature ≤ 18°C, salinity ≤ 36.0); coastal water (CW, temperature ≥ 20°C, salinity ≤ 35.4); and tropical water (TW, temperature ≥ 20°C, salinity ≥ 36.0).

### Data Analysis

Comparisons of plankton community attributes (abundance of individual taxa, richness, copepod diversity and evenness, and sestonic biomass, in addition to phytoplankton biomass) between sampling areas (oceanic and coastal) were performed with unpaired *t*-tests or the Mann–Whitney rank test when the data did not present equal variance and normality (*Shapiro–Wilk*) (Zar, 2010). The structure of the copepod community was described using the Shannon diversity index H′ (Shannon, 1948), and evenness was calculated according to Pielou (1977).

Multivariate analyses included the following: (i) non-metric multidimensional scaling (nMDS) based on the Bray-Curtis distance matrix of the ln (x + 1) transformed biological data; (ii) a permutational multivariate analysis of variance (PERMANOVA) (Anderson, 2001) based on the same distance matrix and considering two spatial groups (coastal and oceanic) and a total of 999 permutations, to test whether the mesozooplanktonic community was similar in the two areas considered; and (iii) a similarity percentages analysis (SIMPER) based on two groups (coastal and oceanic) to identify the most important species in terms of their contribution to the dissimilarity among the coastal and oceanic areas (Clarke and Warwick, 1994). These analyses were performed using PRIMER 6 + PERMANOVA (Clarke and Gorley, 2001).

Indicator value analysis (IndVal) was performed to estimate the fidelity and specificity of the species in the coastal and oceanic groups. The statistical significance of the indicator values for the species was evaluated using a Monte Carlo permutation test (1000 permutations) (Dufrêne and Legendre, 1997). The indicator value index (IndVal) is based only on within-species comparisons of abundance and occurrence and has been used to express the importance of species as ecological indicators in community classifications (Legendre and Legendre, 1998). The values ranged from 0% (no indication) to 100% (perfect indication). The level of significance was set at *p* < 0.05, and indicator value indices (IndVal) of more than 60% were considered. To calculate IndVal, the program PC-ORD, Version 6.08, from the MJM software (1995–2011) was used. Because each IndVal is calculated independently of other species assemblages, comparisons of indicator values can be performed between taxonomically unrelated taxa, taxa in different functional groups, and taxa in different communities. Comparisons across taxa are sensitive to differences in abundance that may be due to differences in sampling methods (Legendre and Legendre, 1998).

A literature review of the functional traits of marine copepods was carried out, resulting in a matrix for assessing functional traits (based mainly on Benedetti et al., 2016). The following traits were included: (i) average adult female body length (mm), measured with an ocular micrometer under microscope (cephalotorax’s length from 10 to 30 specimens of each specie); (ii) feeding type (active ambush feeding, passive ambush feeding, filter feeding, cruise feeding, mixed feeding); (iii) trophic group (herbivore, carnivore, omnivore, detritivore, herbivore-omnivore, omnivore-carnivore, detritivore-carnivore); (iv) mode of reproduction (broadcat-spawner, sac-spawner); (v) vertical distribution preference (epipelagic, mesopelagic, bathypelagic); (vi) diel vertical migration behavior (Yes, No); and (vii) habitat type (nearshore, shelf break, oceanic). These traits were selected because they are functionally important, describing the life history and ecology of species, and because they are expected to remain more or less constant through time and space (Pomerleau et al., 2015). The Sorensen index was applied to a presence/absence species-by-trait matrix, and the groups that were formed were identified with a cluster dendrogram generated using the average linkage method. The Calinski and Harabasz (1974) criterion was employed to fix the number of functional groups. A SIMPER analysis was performed to determine which characteristics made the greatest contributions to the groups.

## Results

### Hydrology

**FIGURE 2:**
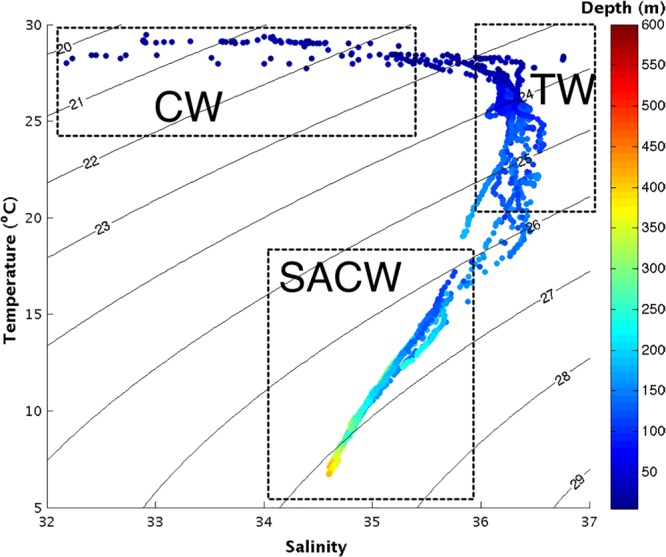
T-S diagram with pairs of temperature and salinity for the whole studied area in September 2014. The colors represent the depth (in meters) at which the pairs of temperature and salinity were obtained. The dashed rectangles identify the water masses. CW, Coastal Water; TW, Tropical Water; SACW, South Atlantic Central Water.

In this study, three water masses were recorded: coastal water (CW), characterized by high temperature and low salinity; tropical water (TW), which is relatively warm and salty; and South Atlantic Central Water (SACW), with low temperatures and salinities between 34.5 and 36. CW forms over the inner portion of the continental shelf as a result of non-isopycnal mixing between river runoff water, estuarine plumes, SACW and TW. Due to the influence of the continental discharge, CW exhibits low salinity (below 35.4), and its temperature is subject to spatial and seasonal variation, exhibiting values between 27°C and 30°C in the present study. CW was mainly restricted to the western side of the river mouth, at stations 10–16, in the 50 m of water depth, possibly being transported westward by the North Brazil Current (NBC). The presence of CW at stations 10–12 was probably due the retroflection of the NBC (**Figure 2**).

TW was present mainly at stations 5–13, on outer continental shelf, was confined to depths between 50 and 200 m of water depth, and exhibited temperatures between 20°C and 27°C and salinities varying from 36 to 36.5. SACW was also found at stations 5 to 13, from 150 to 450 m, and displayed temperatures between 7°C and 18°C and salinities from 34.5 to 36 (**Figure 2**).

**FIGURE 3:**
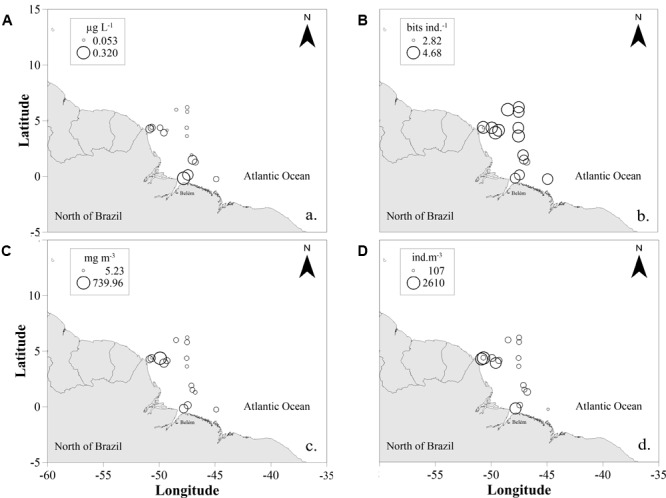
Spatial distribution of surface chlorophyll-a in μg L^-1^ **(A)**, copepod diversity in bits ind^-1^ **(B)**, zooplanktonic biomass in mg m^-3^ **(C)**, and zooplanktonic total density in ind m^-3^ **(D)** in the studied area in September 2014. The area of the circles is proportional to the value of each represented variable.

The chlorophyll-a concentration varied from 0.05 μg L^-1^ (St. 13) to 0.32 μg L^-1^ (St. 1), with average of 0.10 ± 0.07 μg L^-1^ (**Figure 3A**). The general pattern observed was that high values occurred under the influence of the Amazon plume at coastal stations, while lower values were observed at oceanic stations.

### Composition and Frequency of Occurrence

A total of 179 taxa, consisting of Radiozoa, Foraminifera, Cnidaria, Mollusca, Annelida, Nemertea, Crustacea, Bryozoa, Echinodermata, Chaetognatha, and Chordata (**Tables 1, 2**), were identified among the zooplankton. Holoplankton contributed 75% of the zooplankton community, and the copepods were by far the most abundant taxa. A total of 92 species of copepods were recorded, 65% of which were calanoids.

**Table 1:** List of zooplankton taxa (without Copepoda) in the Amazon mouth reef system and adjacent areas (estuarine plume and oceanic system) of Brazil, in September/2014.

**Radiozoa**	**Decapoda**
Spumellaria	Sergestidae (protozoeae)
**Foraminifera**	***Belzebub faxoni* Borradaile, 1915 (adults and mysis)**
*Globigerinoides ruber* (d’Orbigny, 1839)	*Belzebub* sp. (protozoeae)
*Trilobatus trilobus* (Reuss, 1850)	Carideae (larvae)
*Trilobatus sacculifer* (Brady, 1879)	Alpheidae (larvae)
*Globigerinoides* spp.	Hyppolytidae (larvae)
*Globorotalia menardii* (Jones e Brady, 1865)	Axiidae (larvae)
*Globorotalia* spp.	Penaeidae (larvae)
*Tretomphalus bulloides* d’Orbigny, 1826	*Sicyonia* spp. (larvae)
**Cnidaria**	Palaemonidae (phyllosoma)
Hydrozoa	Callianassidae (larvae)
*Liriope tetraphylla* (Chamisso and Eysenhardt, 1821)	Porcellanidae (larvae)
*Aglaura hemistoma* (Peron and Lesueur, 1810)	Paguridae larvae (*Parapagurus* sp.)
Siphonophorae	Brachyura (zoeae and megalopa)
*Chelophyes appendiculata* (Eschscholtz, 1829)	Mysidacea
*Chelophyes contorta* (Lens and van Riemsdijk, 1908)	Isopoda (Epicaridea - larvae)
*Diphyes bojani* (Eschscholtz, 1825)	Amphipoda
*Diphyes dispar* Chamisso & Eysenhardt, 1821	Hyperiidae
*Eudoxoides spiralis* (Bigelow, 1911)	**BRYOZOA (cyphonauta of *Membranipora* sp.)**
*Eudoxoides mitra* (Huxley, 1859)	**CHAETOGNATHA**
*Lensia* spp.	*Ferosagitta hispida* (Conant, 1895)
*Sulculeolaria* sp.	*Serratosagitta serratodentata* (Krohn, 1853)
*Abylopsis tetragona* (Otto, 1823)	*Parasagitta tenuis* (Conant, 1896)
*Bassia bassensis* (Quoy and Gaimard, 1834)	*Parasagitta* spp.
Anthozoa (larvae)	*Pterosagitta draco* (Krohn, 1853)
**Mollusca**	*Flaccisagitta enflata* (Grassi, 1881)
Thecosomata	*Flaccisagitta* spp.
*Cavolinia* spp.	*Krohnitta pacifica* (Aida, 1897)
*Creseis virgula* Rang, 1828	**ECHINODERMATA (pluteus, bipinaria, auricularia, brachiolaria)**
*Creseis clava* Rang, 1828	**CHORDATA**
*Creseis* spp.	Appendicularia
*Lamellaria* (larvae)	*Appendicularia sicula* Fol, 1874
Gastropoda other (veliger/adult)	*Oikopleura (Vexillaria) dioica* (Fol,1872)
Bivalvia (veliger/juvenile)	*Oikopleura (Coecaria) longicauda* (Vogt, 1854)
**Annelida**	*Oikopleura* spp.
Polychaeta larvae (different stages)	*Fritilaria* spp.
**Nemertea (larvae)**	Thaliacea
**Crustacea**	*Thalia cicar* (Forskal, 1775)
Ostracoda	*Thalia* spp.
*Euconchoecia* sp.	*Salpa* sp.
Cirripedia	*Iasis cylindrica* Cuvier, 1804
*Balanus* (cypris)	Doliolidae (*Dolioletta* sp.)
Stomatopoda (larvae Erichthus and Alima)	Vertebrata
Euphausiacea (adults and furcilia, calyptopis)	Teleostei (egg)
	Teleostei (larvae)

**Table 2:** List of copepod species in the Amazon mouth reef system and adjacent areas (estuarine plume and oceanic system) of Brazil, in September/2014.

*Mesocalanus* sp.	*Lucicutia flavicornis* (Claus, 1863)^O^
*Nannocalanus minor* (Claus, 1863)^O^	*Lucicutia* spp. (juvenil)
*Neocalanus gracilis* Dana, 1849^O^	*Candacia pachydactyla* (Dana, 1848)^O^
*Neocalanus robustior* (Giesbrecht, 1888)^O^	*Candacia bispinosa* (Claus, 1863)^O^
*Neocalanus* spp.	*Candacia curta* (Dana, 1849)^O^
*Undinula vulgaris* (Dana, 1849)^N^	*Candacia truncata* (Dana, 1849)^O^
*Subeucalanus subtenuis* (Giesbrecht,1888)^O^	*Candacia* spp. (juvenil)
*Subeucalanus pileatus* (Giesbrecht, 1888)^O^	*Pontellina plumata* (Dana, 1849)^O^
*Subeucalanus* spp.	*Pontellopsis* spp.
*Pareucalanus sewelli* (Fleminger, 1973)^O^	*Labidocera fluviatilis* F. Dahl, 1894^N^
*Rhincalanus cornutus* (Dana, 1849)^O^	*Labidocera* spp.
*Paracalanus aculeatus* Giesbrecht, 1888^O^	*Calanopia americana* F. Dahl, 1894^N^
*Paracalanus quasimodo* Bowman, 1971^N^	*Acartia (Odontacartia) lilljeborgi* Giesbrecht, 1889^NE^
*Paracalanus nanus* Sars G.O., 1925^N^	*Acartia (Acartia) danae* Giesbrecht, 1889^O^
*Paracalanus* spp. (juvenil)	*Acartia (Acanthacartia) tonsa* Dana, 1849^E^
*Parvocalanus crassirostris* (Dahl, 1894)^N^	*Acartia* spp. (juvenil)
*Acrocalanus longicornis* Giesbrecht, 1888^O^	*Oithona tenuis* Rosendorn, 1917^NE^
*Acrocalanus gracilis*^O^	*Oithona nana* Giesbrecht, 1892^NE^
*Acrocalanus* spp. (juvenil)	*Oithona plumifera* Baird, 1843^ON^
*Calocalanus pavo* (Dana, 1849)^O^	*Oithona pseudofrigida* Rosendorn, 1917^NE^
*Calocalanus pavoninus* Farran, 1936^O^	*Oithona setigera* (Dana, 1849)^ON^
*Calocalanus tenuis* Farran, 1926^O^	*Oithona hebes* Giesbrecht, 1881^NE^
*Calocalanus styliremis* Giesbrecht, 1888^O^	*Oithona* spp.
*Mecynocera clausi* Thompson, 1888^O^	*Oncaea media* Giesbrecht, 1891^O^
*Eucalanus* sp.	*Oncaea venusta* Philippi, 1843^ON^
*Clausocalanus furcatus* (Brady, 1883)^O^	*Oncaea mediterranea* (Claus, 1863)^O^
*Clausocalanus mastigophorus* (Claus, 1863)^O^	*Oncaea scottodicarloi* Heron & Bradford-Grieve, 1995^O^
*Clausocalanus* spp.	*Oncaea* spp.
*Aetideus giesbrechti* (Cleve, 1904)^O^	*Sapphirina ovatolanceolata gemma* Lehnhofer, 1929^O^
*Euchaeta marina* (Prestandrea, 1833)^O^	*Sapphirina nigromaculata* Claus, 1863^O^
*Euchaeta* spp. (juvenil)	*Sapphirina iris* Dana, 1849^O^
*Scolecithrix danae* (Lubbock, 1856)^O^	*Copilia quadrata* Dana, 1849^O^
*Scolecithrix* sp.	*Copilia mirabilis* Dana, 1849^O^
*Haloptilus longicirrus* Brodsky, 1950^O^	*Copilia lata* Giesbrecht, 1891^O^
*Haloptilus* spp.	*Corycaeus (Corycaeus) speciosus* Dana, 1849^O^
*Heterorhabdus* sp.	*Ditrichocorycaeus amazonicus* F. Dahl, 1894^N^
*Temora stylifera* (Dana, 1849)^ON^	*Urocorycaeus lautus* Dana, 1849^O^
*Temora turbinata* (Dana, 1849)^ON^	*Corycaeus* spp.
*Temora* spp.	*Onychocorycaeus giesbrechti* (F. Dahl, 1894)^O^
*Pleuromamma xiphias* (Giesbrecht, 1889)^O^	*Onychocorycaeus latus* (Dana, 1849)^O^
*Pleuromamma* spp.	*Onychocorycaeus agilis* (Dana, 1849)^O^
*Centropages violaceus* (Claus, 1863)^O^	*Farranulla gracilis* (Dana, 1849)^O^
*Centropages longicornis* Mori, 1932^ON^	*Farranulla* spp.
*Centropages velificatus* (Dana, 1849)^ON^	*Microsetella rosea* (Dana, 1847)^O^
*Centropages* spp.	*Macrosetella gracilis* (Dana, 1847)^O^
*Pseudodiaptomus* spp.	*Euterpina acutifrons* Dana, 1847^O^

*O, oceanic; N, neritic and E, estuarine (according to Björnberg, 1963, 1981; Bradford-Grieve et al., 1999).*

Common taxa with a wide distribution in the area (frequency of occurrence > 75%) were the copepods *Clausocalanus furcatus, Nannocalanus minor, Oithona plumifera, Corycaeus (Corycaeus) speciosus, Oncaea media* and *Farranula gracilis* and the chaetognath *Flaccisagitta enflata*, the appendicularian *Oikopleura (Vexillaria) dioica*, and Hydrozoa and Polychaeta (larvae). These taxa comprised 5.8% of the mesozooplankton community. In the study area, 14.8% of the taxa in the community were frequent (45–74% frequency of occurrence), whereas 31.8% of the taxa in the community presented a frequency of occurrence between 15 and 44%. The rare taxa (<14% frequency of occurrence) accounted for 47.6% of the mesozooplankton community. Copepod species represented nearly 50% of the rare taxa.

### Diversity and Abundance

High copepod species diversity values were found in all stations (**Figure 3B**), varying from 2.09 bits ind^-1^ (St. 17) to 4.15 bits ind^-1^ (St. 14), with a mean value of 3.37 ± 0.56 bits ind^-1^. Higher values were registered in oceanic samples, with no significant differences among areas (*t*-test; *p* = 0.213). Evenness was also very high, varying from 0.57 (NB6) to 0.86 (St. 16), and mean value of 0.72 ± 0.08, with no significant differences between oceanic and coastal samples (*t*-test; *p* = 0.648).

Total sestonic biomass varied from 5 mg m^-3^ (NB1) to 73.8 mg m^-3^ (NB2), with an average of 13 ± 18 mg m^-3^ (**Figure 3C**). The highest biomass values were caused by high abundances of the appendicularians *Oikopleura* spp. and chaetognaths *Flaccisagitta* spp., followed by the copepods *Oithona plumifera, Clausocalanus furcatus* and Paracalanidae. Additionally, we observed a large amount of other elements in several samples, such as aggregates represented mainly by appendicularians houses (∼10–40% of the non-zooplanktonic organisms; data not shown), in addition to algae fragments, other detritus, microplastics and few fine sediments. In all samples with high appendicularian abundances, we detected high contribution of their houses.

The total zooplankton density varied from 107.98 ind. m^-3^ (NB10) to 2,609.24 ind. m^-3^ (St. 17) with a mean of 780.46 ± 830.95 ind. m^-3^ (**Figure 3D**). Significant differences were found between coastal and oceanic samples to the copepods *Oithona plumifera, Clausocalanus furcatus* and Paracanalidae (*p* = 0.03).

Copepods represented 60% of total community density, followed by appendicularians (24%), considering all stations together. At each station, copepods accounted for > 58%, except for St.16 (28.1%) and St.14 (49.4%). At these stations, appendicularians (mainly, *Oikopleura*) comprised 63.1 and 28.9% of the community density, respectively.

### Multivariate and Indicator Species Analyses

**FIGURE 4:**
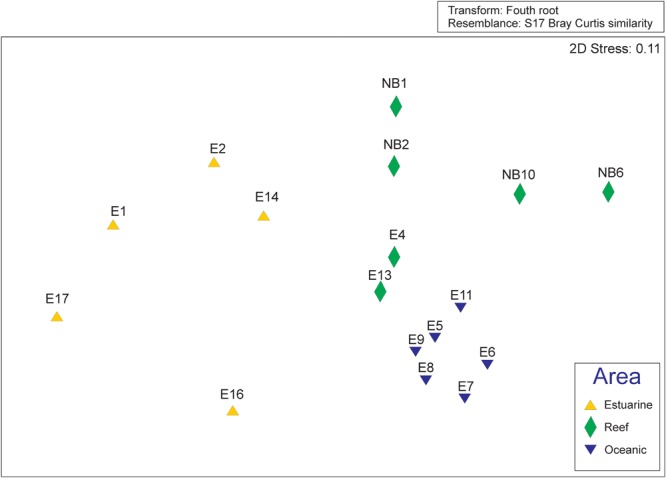
Ordination of the non-Metric Multidimensional Scaling based on the Bray-Curtis distance matrix calculated on the fourth root transformed biological data matrix. The colors and shapes of the points distinguish the area at which the samples were collected according to the legend displayed within the figure.

MDS showed clear separation of the oceanic and coastal samples, and in coastal samples, clear separation was observed between the reef area and other coastal areas (**Figure 4**). A cluster group was formed by the non-reef samples from coastal zones, and another group was formed for oceanic and reef samples, which were separated into two subgroups (data not shown). A significant source of spatial variation in the taxonomic composition of the mesozooplankton community was suggested by the PERMANOVA result (*p* = 0.001). The observed differences were examined based on the average dissimilarity (SIMPER), and a total of 42 species contributed 70% of this spatial dissimilarity. These differences were caused by varying abundance among the following dominating species at each sampling site: *Clausocalanus* spp. (juvenile), *Oikopleura (Vexillaria) dioica, Euterpina acutifrons, Undinula vulgaris, Parvocalanus crassirostris*, Crustacea (nauplius) and *Calocalanus pavo*, contributing 18.6% to the observed dissimilarity.

The Indicator Value index showed three groups of indicators (**Table 3**). The first was the strongest and was indicative of coastal waters under the influence of the estuarine plume, where the best indicator taxa were *Euterpina acutifrons* (99.2, *p* = 0.0002), Hydromedusae (94, *p* = 0.0008), *Oikopleura (Vexillaria) dioica* (93.9, *p* = 0.0018), *Parvocalanus crassirostris* (92.9, *p* = 0.038) and Nauplius (81.3, *p* = 0.0426). These taxa highlight the great relevance of the riverine outflow in the area. The oceanic species group was characterized by *Clausocalanus* spp. (juvenile) (96.2, *p* = 0.0002) and the reef system group by *Oithona plumifera* (63.4, *p* = 0.046).

**Table 3:** Observed randomized indicator groups along the Amazon coast, in September/2014.

Taxa	Value (IV)	Mean	*SD*	*p*^∗^
**Group 1 - Coastal/estuarine plume**				
Polychaeta (larvae)	57.1	46.7	9.94	0.1586
*Corycaeus (Corycaeus) speciosus*	63.4	49.9	11.89	0.1484
*Oikopleura (Vexillaria) dioica*	**93.9**	52.7	14.22	0.0018
Hydromedusae	**94.0**	45.6	12.49	0.0008
Gastropoda (veliger)	29.8	41.9	11.26	0.8806
*Parvocalanus crassirostris*	**92.9**	61.4	15.59	0.0384
Crustacea (nauplius)	**81.3**	51.9	15.10	0.0426
Chaetognatha	62.4	41.8	11.46	0.0590
*Paracalanus aculeatus*	68.6	42.7	14.29	0.0600
Euphausiacea	13.1	56.7	16.23	1.0000
*Oithona hebes*	24.9	45.9	15.76	0.9092
*Acrocalanus longicornis*	43.6	34.0	12.66	0.2100
*Euterpina acutifrons*	**99.2**	39.3	15.39	0.0002
*Oithona nana*	35.6	39.3	14.74	0.5655
Paracalanidae	35.8	38.1	14.66	0.4599
*Dolioletta* sp.	37.5	41.1	15.19	0.5895
**Group 2 - Oceanic**				
*Oncaea media*	28.6	49.4	11.19	0.9866
*Oikopleura longicauda*	47.9	51.0	14.87	0.4743
*Undinula vulgares*	43.3	43.6	12.36	0.4323
*Oncaea venusta*	51.9	38.0	10.29	0.1102
*Rhincalanus cornutus*	25.8	36.8	12.57	0.8214
*Scolecitrix danae*	37.6	35.7	11.97	0.3737
*Clausocalanus* sp.	**96.2**	34.4	12.63	0.0002
**Group 3 - Reef**				
*Clausocalanus furcatus*	57.7	47.7	9.26	0.1800
*Farranula gracilis*	53.9	46.2	10.43	0.2374
*Nannocalanus minor*	32.1	45.2	11.14	0.9034
*Oithona plumifera*	**63.4**	42.7	10.42	0.0462
*Flaccisagitta enflata*	40.4	47.3	12.42	0.6707
*Macrosetella gracilis*	37.6	38.7	12.48	0.4711
*Paracalanus quasimodo*	44.7	37.9	11.94	0.2370
Cirripedia (cypris)	52.3	41.7	15.07	0.2396
*Euchaeta marina*	32.7	35.5	13.06	0.4931
*Temora stylifera*	26.5	34.2	12.36	0.6915
**Averages**	**51.69**	**43.49**	**12.89**	**0.3786**

*^∗^Proportion of randomized trials with an indicator value equal to or exceeding the observed indicator value. *p* = (1 + number of runs ≥ observed)/(1 + number of randomized runs). Bold values = indicator species.*

### Copepod Functional Traits

**FIGURE 5:**
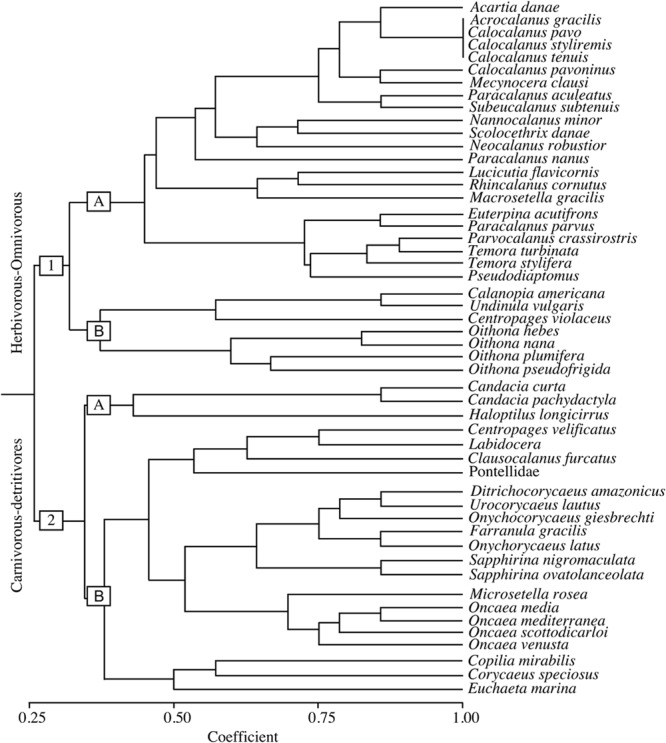
Dendrogram of the copepods trait groups identified in the study area in September 2014. The numbers 1 and 2 represent the main groups (Herbivorous-Omnivorous, and Carnivorous-Detritivores, respectively) and “a” and “b” represent further subdivisions of these groups according to feeding and reproductive strategies, size characteristics, distance from the coast and vertical migration patterns.

The trophic strategy was the major functional trait related to the sorting of the community, forming two main groups: an herbivorous-omnivorous group (group 1) and a carnivorous-detritivorous group (group 2) (**Figure 5**). These groups were each subdivided into two subgroups, according to feeding and reproduction strategies, size, distance from the coast and migration.

Subgroup 1a was the largest group and was mostly comprised of oceanic or coastal copepods, filterers, broadcasters, non-migrant taxa and taxa with smaller sizes (500–1000 μm). This group consisted mainly of calanoids, such as those of the genera *Acartia, Calocalanus, Temora, Parvocalanus*, and *Paracalanus*, and the coastal harpacticoid *Euterpina acutifrons.* Subgroup 1b essentially consisted of *Oithona* spp. and common calanoids of the *Calanopia, Undinula* and *Centropages* genera, which are characterized by a neritic-coastal-estuarine distribution, active ambush behavior, weak diel vertical migration and a small size (1500–2500 μm).

Subgroup 2a included only three species of calanoids belonging to the *Candacia* and *Haloptilus* genera, which are typically oceanic, epimesopelagic and relatively large in size (2500–6500 μm). Subgroup 2b was the second largest group and was divided in two groups: group 2.b.1 was mainly composed of cyclopids of the families Oncaeidae, Corycaeidae and Sapphirinidae, in addition to two important calanoids (*Clausocalanus furcatus* and *Centropages velificatus*). These cyclopids are oceanic, omnivorous or carnivorous, cruiser, sac-spawner, non-migrant, epimesopelagic species. The two calanoids are filterers, broadcasters and non-migrants and are relatively small in size. Group 2.b.2 was composed of three common, but low-abundance copepods [*Euchaeta marina, Corycaeus (Corycaeus) speciosus* and *Copilia mirabilis*], all which are oceanic, omnivorous or carnivorous species that exhibit cruising or active ambush behavior, weak to strong vertical diel migration, an epipelagic habit and variable class sizes (1500–4500 μm).

## Discussion

### Mesozooplankton and Copepod Functional Traits

The Amazon area showed a mixture of communities from estuary, coastal, reef and oceanic habitats, where the predominance of holoplanktonic species, mainly copepods (with 92 species), was the rule. The high species richness, diversity and evenness of copepods, with no significant differences between samples, can be explained by the habitat heterogeneity hypothesis (e.g., MacArthur and Wilson, 1967), in which physically complex habitats may offer more niches and diverse means of using the available resources and therefore increase species diversity (Bazzaz, 1975). The predominance of copepods, in terms of richness, in this area was previously described by Calef and Grice (1967) that mentioned 150 species, while Melo (2006) registered 109 species, among which 97 species were common to the work of Calef and Grice (1967). Most of these species were also previously seen in the Brazilian Northeast (Neumann-Leitão et al., 1999, 2008, Gusmão, 2000) and Southeast (Miyashita et al., 2009, Melo et al., 2016). Other important holoplanktonic groups were appendicularians [mainly, *Oikopleura (Vexillaria) dioica*], chaetognaths (*Flaccisagitta enflata* and *Serratosagitta serratodentata*), and decapods of the family Luciferidae (*Belzebub faxoni*). These groups were frequently found at high abundances. On the other hand, meroplankton was rare, and few groups were registered.

The MDS showed a clear separation in the communities along the salinity gradient in the plume. The oceanic samples differ from coastal ones, and this last were separated in estuarine plume and reef samples. This gradient was also observed to phytoplankton assemblages by Goes et al. (2014). The IndVal analysis corroborated the MDS and discriminate the indicator species of the three areas. Typical euryhaline species as *Euterpina acutifrons, Oikopleura (Vexillaria) dioica* and *Parvocalanus crassirostris* characterized the estuarine community, while *Clausocalanus* spp. (juvenile) and *Oithona plumifera* characterized oceanic and reef, respectively. *Oithona plumifera* was abundant, especially over the reef area and the region of retroflection, and predominates in oligotrophic waters of the tropical Atlantic (e.g., Webber and Roff, 1995), and is exceptionally adorned with spikes in form of feathers on its first anthem, the thoracic legs, and the urosoma, including the caudal branch (Giesbrecht, 1892). This richness of ornaments, which are potentially sensory structures, suggests that this species quickly recognizes hydrodynamic signals from the surrounding environment (Paffenhöfer, 1998), potentially imparting an advantage in an area with complex hydrodynamic processes.

As the prominent group in zooplankton community from the reef system under the Amazon plume influence the coastal and oceanic copepods *Clausocalanus furcatus, Nannocalanus minor, Oithona plumifera, Corycaeus (Corycaeus) speciosus, Oncaea media* and *Farranula gracilis* stood out among the zooplankton in terms of their frequency of occurrence, whereas in terms of abundance, the coastal copepod species *C. furcatus, Parvocalanus crassirostris, O. hebes* and *Euterpina acutifrons* were the most important. These last three species are characteristic of estuarine systems in Brazil (Björnberg, 1981), and were the main indicators in the studied area, showing the expressive importance of the estuarine influence of the Amazon River. On the other hand, the coastal-oceanic species *Undinula vulgaris, Temora turbinata, O. plumifera* and *O. media* contributed sporadically in terms of abundance and have been previously recorded as dominant in the samples from the mouth of the Amazon River, in an area slightly north of our study area (Calef and Grice, 1967).

In the more oceanic stations, the common coastal copepods *C. furcatus, Nannocalanus minor, O. plumifera, C. speciosus, O. media* and *F. gracilis* showed higher densities, which can be explained by the hydrographic regime, conditioned by the Northern Brazil Current (NBC) driven by trade winds prevailing in the region during almost the whole year (Castro and Miranda, 1998). From January to June, the plume of the Amazon River north of 5°N is primarily located west of 52°W, extending toward the Caribbean, and from August to October, approximately 70% of the water in the Amazon River plume is carried eastward via retroflection of the North Brazil Current, while the remaining 30% is diverted northwest toward the Caribbean (Lentz, 1995). The retroflection of the NBC carries a nutrient-enriched water mass with varying salinities and temperatures in the subsurface layer (varying between 23.5 and 35.9 and between 28.3 and 28.9°C, respectively) (Paiva, 2002). This fertilization of oceanic waters by retroflection of the North Brazil Current allows a denser zooplanktonic community to be sustained that is composed of common coastal pelagic copepods.

Other zooplankton groups were also abundant at some stations and showed a wide distribution, such as the chaetognaths and decapods. All of the species recorded in this group are predominantly predators of the zooplanktonic community. Their diet consists mainly of copepods, exerting a considerable influence on the structure of lower trophic levels (Pearre, 1980). Additionally, the holoplanktonic decapod *Belzebub faxoni* (cited as *Lucifer faxoni*) was found to be common in studies conducted in coastal areas of the Amazon (Melo N.F.A.C. et al., 2014; Conroy et al., 2016). According to Longhurst and Pauly (1987), this decapod is an important species in neritic tropical plankton, where salinity is lower, as is the case for the Amazon plume.

The copepod functional groups identified in the present study highlight the connections of species through their ecological functions. Two main functional groups of copepods occurred in the area, mainly in the reef one, where the Amazon plume dominates. The main factor separating the groups was their trophic strategies, which are commonly regulated by female weight, environmental temperature, and food (Blaxter et al., 1998, Bunker and Hirst, 2004). In terms of feeding, three strategies have developed in copepods: (1) capturing prey by straining water (filtering); (2) wait for prey to pass by and then attacking it (active ambushing); and (3) capturing prey that arrives at the predator via the movement of the water (cruising) (Kiørboe and Sabatini, 1995). The species that comprised group 1 were mostly oceanic (although some dominant neritic-coastal-estuarine species also occurred), omnivore-herbivore, filtering, small-sized, epipelagic, non-migrant and broadcaster species. This group consisted mainly of estuarine-coastal occurrence genera (*Oithona, Acartia, Temora, Parvocalanus, Paracalanus* and *Euterpina*). Group 2, in contrast, was represented by copepods with an oceanic distribution that were carnivorous and detritivorous, exhibiting cruising or active ambushing habits, larger sizes and weak to strong migration habits, behaving as sac-spawners or broadcasters (e.g., *Candacia, Euchaeta* and *Haloptilus* genera, and the cyclopids of the families Oncaeidae, Corycaeidae and Sapphirinidae).

However, we found a large number of copepod species coexisting with apparently similar function traits in this reef area. Also in line with our findings, Hayward and McGowan (1979) found a coexistence of a large number of species of copepods in tropical and subtropical waters in the North Pacific gyre and no differences in the stomach contents of several of these species was verified. In accordance with our hypotheses, the trait composition of copepods in the reef system was mainly characterized by omnivorous species of small size, i.e. typical species of estuarine ecosystems. According to some studies (Turner, 2004, Calbet, 2008), the observed trophic regimes are diverse, with most species being omnivorous and the rest predominantly herbivorous, carnivorous, or detritivorous. On the other hand, in oceanic areas, an increase of large and carnivorous copepods with increasing occurrence of those species that exhibit cruising or active ambush behavior, and weak to strong vertical diel migration, was seen. As expected, we found larger calanoids in oceanic stations than in coastal-estuarine ones, which support findings of prior studies (Neumann-Leitão et al., 1999, 2008, Miyashita et al., 2009). Even the results having showed a common functional structure of the copepod community (e.g., Benedetti et al., 2016), future researches should elucidate in more details the ecological role of such species to the Amazon reef benthic and pelagic communities, which could help to assess the impact of our findings.

### Mesozooplankton and the Implications to the Amazon Reef System

In our study, the general pattern of chlorophyll-a and sestonic biomass was of high values under the influence of the Amazon plume at coastal stations, including those under the reef area, while lower values were observed at oceanic stations. Araujo et al. (2017) highlight the productive characteristic in the Amazon plume in comparison to oceanic adjacent area. Similar observations were made by Subramaniam et al. (2008) that affirm the river plume contribution as a nutrient provider to enhance primary production. This spatial shift in production is transferred through the food web, as emphasized by Conroy et al. (2016), that observed a dominance transition from meso- to microzooplankton grazers between coastal to oceanic area, reflecting meso- and oligotrophic conditions, respectively.

The Amazon River drains 1 million cubic meters of water per year and exhibits the largest estuarine plume in the world (Nittrouer et al., 1991), presenting high turbidity, due to a sediment load of approximately 11 to 13.10^8^ tons per year (Kineke et al., 1996), and low salinity, extending up to 200 km (Geyer and Kineke, 1995). This dynamic system provides a combination of plankton and fine sediments, and due to the extreme turbidity of the surface layer, the penetration of light is reduced. Therefore, differences in surface and sub-surface layers, as habitats for phytoplankton, usually result in low cell numbers. According to the ”cascade effect,” it is expected that the abundance of mesozooplankton should be also affected. However, appendicularians and small copepods were frequent and abundant, and exhibited high contribution percentage among the zooplankton at some stations. This finding could be attributed to the capability of appendicularians to use picoplankton and smaller nanoplankton particles, or even colloidal materials, as a food source, benefiting their population in this pelagic system due to the large supply of fine materials from the Amazon River plume. Additionally, some small cyclopids can use small particles aggregated on settling appendicularian houses (e.g., Turner, 2004). Although the low-salinity plume stays well above the seafloor, the plume may also interact dynamically with benthic organisms through particle flux, shear, and enhanced eddy stirring and mixing (Coles et al., 2013). Our results are in agreement with these patterns, and the isotopic signatures for Amazon Rivers, mangrove waters, surface Atlantic waters, and deep Atlantic waters reinforce the contribution from terrestrial and mangrove-derived material to the reefs’ DOC and POC pools (Moura et al., 2016).

These results highlight the importance of the pelagic realm to the reef system, supporting a complex and rich zooplankton community, including the probable sinking of *Oikopleura* mucous nets clogged with food particles (Alldredge et al., 1998, Hopcroft et al., 1998, Berline et al., 2011), which may contribute to maintaining this system.

Finally, in the northern Brazil where the Amazon River joins the saline water of the continental shelf, low salinity and turbid water masses form a plume that persists both temporally and spatially. The turbid estuarine plume of Amazonas extends for many kilometers, covering an expansive area and carrying many zooplanktonic organisms, proved by some estuarine-coastal species occurring in stations far from the Amazon mouth and the coast. This turbidity is due to the resuspension of sediments from the bottom due to wind, tides and convective mixing caused by the discharge of rivers. Despite the high levels of particulate matter, their effect on suspension feeding zooplankton of the pelagic system in the Amazon reef area (although it has not been studied extensively) appears to be minimal in terms of mesozooplankton composition and structure (e.g., copepod richness, diversity and functional traits), which are common according to previous studies elsewhere. However, our data shows a clear separation of the oceanic and coastal communities, and in the coastal community, a clear separation exists between the reef area and the other coastal areas, showing that the oceanic region also contributes with several species to the Amazon reef system. This ecosystem shows a mixture of estuarine, coastal and oceanic zooplanktonic communities, with a gradual separation among them along the salinity gradient in the plume.

## Author Contributions

MA, FT, and RM coordinated the projects. SN-L, MA, DV, RS, and RM designed and coordinated the fieldwork. SN-L, PM, MM, and RS wrote the paper. SN-L, DV, RS, MM, PM, XD, AS, LF, RC, NM, AC, FT, and RM contributed with data and data analyses.

## Conflict of Interest Statement

The authors declare that the research was conducted in the absence of any commercial or financial relationships that could be construed as a potential conflict of interest.
